# An *In Vitro* and *In Vivo* Evaluation of a Reporter Gene/Probe System hERL/^18^F-FES

**DOI:** 10.1371/journal.pone.0061911

**Published:** 2013-04-12

**Authors:** Chunxia Qin, Xiaoli Lan, Jiang He, Xiaotian Xia, Yueli Tian, Zhijun Pei, Hui Yuan, Yongxue Zhang

**Affiliations:** 1 Department of Nuclear Medicine, Union Hospital, Tongji Medical College, Huazhong University of Science and Technology, Wuhan, China; 2 Department of Radiology and Medical Imaging, University of Virginia, School of Medicine, Charlottesville, Virginia, United States of America; Medical University Innsbruck, Austria

## Abstract

**Purpose:**

To evaluate the feasibility of a reporter gene/probe system, namely the human estrogen receptor ligand binding domain (hERL)/16α-[^18^F] fluoro-17β-estradiol (^18^F-FES), for monitoring gene and cell therapy.

**Methods:**

The recombinant adenovirus vector Ad5-hERL-IRES-VEGF (Ad-EIV), carrying a reporter gene (hERL) and a therapeutic gene (vascular endothelial growth factor, VEGF165) through an internal ribosome entry site (IRES), was constructed. After transfection of Ad-EIV into bone marrow mesenchymal stem cells (Ad-EIV-MSCs), hERL and VEGF165 mRNA and protein expressions were identified using Real-Time qRT-PCR and immunofluorescence. The uptake of ^18^F-FES was measured in both Ad-EIV-MSCs and nontransfected MSCs after different incubation time. Micro-PET/CT images were obtained at 1 day after injection of Ad-EIV-MSCs into the left foreleg of the rat. The right foreleg was injected with nontransfected MSCs, which served as self-control.

**Results:**

After transfection with Ad-EIV, the mRNA and protein expression of hERL and VEGF165 were successfully detected in MSCs, and correlated well with each other (R^2^ = 0.9840, *P*<0.05). This indicated the reporter gene could reflect the therapeutic gene indirectly. Ad-EIV-MSCs uptake of ^18^F-FES increased with incubation time with a peak value of 9.13%±0.33% at 150 min, which was significantly higher than that of the control group. A far higher level of radioactivity could be seen in the left foreleg on the micro-PET/CT image than in the opposite foreleg.

**Conclusion:**

These preliminary *in vitro* and *in vivo* studies confirmed that hERL/^18^F-FES might be used as a novel reporter gene/probe system for monitoring gene and cell therapy. This imaging platform may have broad applications for basic research and clinical studies.

## Introduction

Gene and cell therapy holds great promise as a potential treatment for various diseases, especially for ischemic disease and cancer research [1]. However, some problems such as a lack of tracing cells and monitoring gene expression after treatment exist. Currently, PET reporter gene/probe systems are the prevalent molecular imaging strategy for monitoring therapeutic cell and gene expression in terms of magnitude, location and duration, wherein the therapeutic gene is co-expressed together with the reporter gene which enables indirect external monitoring expression of the therapeutic gene [2], [3], [4].

There are two kinds of reporter genes commonly used for radionuclide imaging. One is herpes simplex virus type 1 thymidine kinase (HSV1-tk). The other is based on a membrane associated receptor or transporter, such as the dopamine 2 receptor, somatostatin receptor and sodium iodide symporter (NIS) [5], [6], [7]. However, both kinds of reporter genes come with shortcomings. HSV1-tk is also known as "suicide gene", its application is limited due to the toxic effects of the expression products on normal cells [8]; Membrane associated receptor or transporter may cause a series of physical problems related to the cellular signal transduction [9]. Thus exploration and development of a novel reporter gene imaging system that is safe and effective was an important component of this study. An ideal reporter gene system used for radionuclide imaging should have the following features. First, the reporter gene should be non-toxic, non-immunogenic, non-secretary, small in size, and have no endogenous protein present in the target region. Second, the tracer should be safe for use in humans and be able to combine with the reporting protein effectively after permeating the cell membrane, preferably the blood brain barrier when the tracer is used to study some brain diseases [10].

In the present study, we developed a reporter gene imaging system that uses the human estrogen receptor ligand-binding domain (hERL) as the reporter gene and 16α-[^18^F]-fluoro-17β-estradiol (^18^F-FES) as the radio-ligand. As a reporter gene, hERL has many advantages. First, it is a fragment of the estrogen receptor and has no transcriptional role in estrogen gene regulation due to lack of N-terminal region, avoiding unnecessary physiological role and keeping the feature of estrogen specifically. Second, hERL is a human protein, meaning that it is not or is only minimally immunogenic. In addition, there is very low endogenous expression of estrogen receptor except for the uterus, ovaries and mammary glands. Thus hERL can be used as an exogenous gene and introduced into target cells or tissues without endogenous protein interference. Estrogen receptor is a member of the nuclear hormone family of intracellular receptors [11]. Since estrogen is a steroidal hormone, it can pass through the phospholipid membranes of the cell [12], [13]. When estrogen enters the nucleus, it binds to the estrogen receptor. So estrogen or its analogues, which can recognize and specifically bind to ERL, were selected as the reporter probes to monitor the reporter gene. At present, many radionuclides, such as ^125/131/123^I, ^18^F and ^99m^Tc, can be used to label estrogen or its derivatives [14], [15]. 16α-[^18^F]fluoro-17β-estradiol (^18^F-FES) is a well-established positron emission tomography (PET) tracer with a known labeling procedure, and as a small lipophilic molecule, it has a well-characterized biodistribution and high blood–brain barrier permeability [16], [17], [18], [19], [20]. ^18^F-FES can get internalized into ER expressing cells and bind with ER, then accumulates and traps in the targeted cells. It has already been clinically used for the diagnosis of breast cancer and gynecological carcinomas of the pelvic cavity [21], [22]. All of these characteristics make ^18^F-FES perfect for reporter gene imaging.

In relation to reporter gene imaging, researches mainly focused on cancer, while cell/gene therapy with great potential in many other diseases is rarely involved. Bone mesenchymal stem cells (MSCs) account for a small population of cells in bone marrow. They constitute a non-hematopoietic component with the capacity to differentiate into a variety of cell lineages, including adipocytes, osteocytes, chondrocytes, cardiomyocytes, neuro progenitors, and stromal cells [23]. In addition, MSCs can be readily isolated and expanded in vitro. So MSCs are considered to be a promising platform for cell and gene therapy for a variety of diseases, including tissue/organ damage, inflammation and cancer [24]. In the past decade much progress has been made in the development of gene therapy. Vascular endothelial growth factor (VEGF) is a potent angiogenic cytokine and has been delivered as a recombinant protein, using plasmids or viral gene constructs to encourage blood vessel formation in ischemic tissues [25], [26]. In the present study, MSCs and VEGF165 were used as the therapeutic cell and gene; hERL/^18^F-FES was used as the reporter gene/probe system to monitor the expression of the therapeutic gene and stem cell indirectly in vivo. The aim of our study was to evaluate the feasibility of a new reporter gene/probe system, hERL/^18^F-FES, for monitoring gene and cell therapy, and to provide a theoretical basis for in vivo imaging using animal models of ischemic heart disease.

## Materials and Methods

### Gene Transfer Vector

Recombinant adenoviruses vector Ad5-hERL-IRES-VEGF (Ad-EIV), carrying a reporter gene (hERL) and a therapeutic gene (VEGF165) through an internal ribosome entry site (IRES), was constructed and purified in Vector Gene Technology (Beijing, China). The hERL and VEGF genes in the recombinant vectors were identified using the polymerase chain reaction (PCR). The viral titer of Ad5-hERL-IRES-VEGF was 1×10^9^ TU/ml as determined using the 50% Tissue Culture Infective Dose (TCID50) method.

### Rat Bone Mesenchymal Stem Cell Isolation and Culture

All protocols involving animals were conducted according to the standards of international regulations. Sprague-Dawley rats aged 6–8 weeks and weighing 100–150 g were obtained from the Experimental Animal Center of Tongji Medical College, Huazhong University of Science and Technology (Wuhan, China). They were euthanized with an overdose of chloral hydrate solution. MSCs were harvested and cultured as described previously [27], [28]. Briefly, both tibias and femurs were dissected free, the ends of the bones were then cut and the bone marrow was extracted and rinsed in 10 mL of Dulbecco's modified Eagle's medium/Ham's F-12 nutrient mixture (DMEM/F12; Hyclone, Beijing, China) with a syringe. Cells were then washed twice with culture medium (DMEM/F12 containing 15% fetal bovine serum) and planted in 75 cm^2^ flasks. They were incubated at 37 °C in a humidified atmosphere containing 5% carbon dioxide (CO_2_). After 3 days, non-adherent cells were removed by replacing the medium and adherent cells further cultured in culture medium for 4 supplementary days. Cells were trypsinized at 80%–90% confluence using 0.25% trypsin solution, and cell viability was checked using the trypan blue dye exclusion test and replated to fresh flasks.

MSC phenotype was considered positive for CD44 and CD90, and negative for CD34 and CD45 [29]. In order to characterize the phenotype of MSCs, immunocytochemical analyses were performed with CD34 and CD44 antibodies. MSCs at passage three were fixed in 4% paraformaldehyde for 30 min, and then permeabilized with 0.1% Triton X-100 for 15 min. After the cells were washed with phosphate-buffered saline (PBS), they were incubated overnight with primary mouse antibodies directed against either the rat CD34 (ready for use, Maixin, China) or CD44 (1∶200, Zhongshan, China) surface antigens at 4 °C. Mouse IgG was added instead of primary antibodies as an isotype control. Cells were then washed with PBS followed by incubation with a secondary antibody (ChemMateTMEnVision+/HRP) for 45 min at room temperature. The signals were visualized using diaminobenzidine substrate and counterstained using hematoxylin. In addition, for further confirmation, the proportion of CD45− and CD90+ cells was analyzed using flow cytometry. Cells at passage three were used. After trypsinization, 2×10^5^ cells were resuspended in 300 µL of PBS and incubated with antibodies directed against either the CD45 (Anti-Rat CD45.2 FITC; eBioscience) or the CD90 (Anti-Mouse/Rat CD90.1 [Thy-1.1] PE; eBioscience) antigens for 30 min. The cells were then washed with PBS, fixed in CellFix (eBioscience) and analyzed using flow cytometry. Data were generated using the CELLQUEST software. Isotype control antibody containing mouse IgG1 FITC and IgG2 PE was used as a control.

### *In Vitro* Viral Infections

MSCs between passages three and ten were transfected with various Ad-EIV viral titers (multiplicity of infection (MOI) = 0, 25, 50, 75 or 100) according to the aim of the experiment. Take a 6-well-plate and MOI = 100 as an example. About 5×10^5^ MSCs per well were incubated with 1×10^7^ TU of adenoviral (10 µl) diluted in 1 ml of Opti-MEM medium without fetal calf serum (FCS) at 37 °C for 4 h, and then the viral supernatant was removed and replaced with culture medium. The adenovirus-infected cells were called Ad-EIV-MSCs and were used after 48 h of infection. MSCs incubated with Opti-MEM at the same volume served as the control, but without virus transfection.

### Real-Time qRT-PCR

The transcription levels of ER and VEGF genes were quantified by real-time reverse transcription (RT)-PCR. Total RNA was isolated from cells using TRIzol® reagent (Invitrogen, Carlsbad, CA, USA) and cDNA was then synthesized from total RNA using SuperScript™ II Reverse Transcriptase according to the manufacturer's protocols (Invitrogen). Real-time RT-PCR using synthesized cDNA as a template was performed with three pairs of PCR primers (Table 1) and a SYBR green mix (Thunderbird SYBR qPCR Mix; Toyobo, Osaka, Japan). The reaction was done in triplicate. Glyceraldehyde phosphate dehydrogenase (GAPDH) was used as an internal control gene. Relative gene expression of ER and VEGF were analyzed using comparative threshold circle (C_T_) method, which means the amplification fold change of ER or VEGF relative to GAPDH in the transfected cells compared with the control nontransfected cells.

**Table 1:** Primers used for qRT-PCR analysis

Gene		Primer sequence	Length (bp)
**Human VEGF165**	Forward	5′-ATGACGAGGGCCTGGAGTGT-3′	227
	Reverse	5′- ACATTTACACGTCTGCGGATCT-3′	
**Human ER**	Forward	5′-GAAGTGCAAGAACGTGGTG-3′	149
	Reverse	5′-AATGCGATGAAGTAGAGCC-3′	
**Rat GAPDH**	Forward	5′-CGCTAACATCAAATGGGGTG-3′	201
	Reverse	5′-TTGCTGACAATCTTGAGGGAG-3′	

### Immunofluorescence Assay for hERL and VEGF165 Expression

MSCs were transfected with Ad-VIE (MOI = 100) for 48 h. Cells were then fixed with 4% paraformaldehyde and permeabilized with 0.1% Triton X-100 for 15 min. Primary antibodies, monoclonal antibodies to ERα (Santa Cruz, USA) at a dilution of 1∶200 and VEGF (Beyotime, China) at a dilution of 1∶200, were added to the cells and incubated overnight at 4 °C. After washing with PBS for three times, cells were incubated with CY3-conjugated goat anti-mouse or rabbit antibody (1∶50) for 45 min. The cells were then washed with PBS and incubated with hochest33258 for 10 min. All fluorescent staining was visualized using a fluorescence microscope. Rabbit IgG was added instead of primary antibodies as an isotype control.

### Radiolabelling of Estradiol

^18^F-FES was prepared using a conventional ^18^F-FDG module and established procedures were used according to the published references [30], [31], [32]. Fluorine-18 was produced by the ^18^O (p,n) ^18^F reaction on ^18^O-enriched water as target material. The necessary precursor, 3-O-methoxymethyl-16, 17-O-sulfuryl-16-epiestriol (MMSE) was purchased from Huayi Isotopes Co. (Changshu, China). Briefly, the total procedure of ^18^F-FES preparation consisted of three steps as follows: (a) fluorination, (b) hydrolysis, and (c) high performance liquid chromatography (HPLC) purification of the final product. The yield of ^18^F-FES was 50.0±2.35%, and the radiochemical purity was 96.1±0.3% (n = 4).

### Cellular Uptake of ^18^F-FES

ER activity was evaluated by measuring the cellular uptake of ^18^F-FES. For this purpose, Ad-EIV-MSCs (MOI = 100) and control nontransfected MSCs in 24-well plates were incubated at 37 °C with 200 µL of ^18^F-FES (2 µCi/mL) for 30, 60, 90, 120 and 150 min. At the end of the incubation period, the radioactive medium was collected from each well, followed by rinsing three times with PBS. The medium and rinses were combined into a tube for the purpose of counting of extra cellular radioactivity. Subsequently, the cells were lysed with 1 N sodium hydroxide solution and the wells were rinsed three times with PBS. Both cell lysate and rinses were collected into an additional tube for counting of intracellular radioactivity. Radioactivity was measured using a gamma counter and corrected for decay. Triplicate samples were performed for all uptake studies. The uptake rate was calculated according to the following formula: Uptake Rate (%) = Count _intracellular_/(Count _extra cellular_+Count _intracellular_)×100%.

### *In Vivo* Micro-PET/CT Imaging

To assess the feasibility of the reporter gene/probe system hERL/^18^F-FES for monitoring gene and cell therapy in vivo, micro-PET/CT were performed in rats. Four rats were prepared for imaging. For each rat, 3−5×10^6^ Ad-EIV-MSCs (MOI = 100) suspended in 100 µL of PBS were injected intramuscularly into left foreleg, and nontransfected MSCs with same number were injected into the opposite foreleg served as a self-control. Positron emission tomography (PET) imaging was performed on a micro-PET/CT scanner (Siemens Inc, Germany) at 1 day after MSCs injection. The injected dose of ^18^F-FES was 200–300 µCi per animal. Images were obtained at 1 h after intravenous tail vein injection of ^18^F-FES. During imaging, the animals were maintained under 10% chloral hydrate anesthesia (0.3 mL/100 g), and placed in prone position on the bed of the scanner. Two bed positions were acquired.

The images were reconstructed and identical regions of interest (ROIs) were drawn on bilateral foreleg areas of rat images. The counts per pixel per minute were converted to tracer activity (Bq/mL) using a calibration constant obtained from scanning a cylindrical phantom. The ROI counts per mL per min were converted to counts per gram per min (assuming a tissue density of 1 g/mL), and divided by the injected dose to obtain an image ROI-derived tissue uptake index expressed as percent injected dose per gram of tissue (% ID/g).

### Statistical Analysis

Data are expressed as mean ± SD. Group comparisons were performed using a two-tailed Student t test or one-way ANOVA where appropriate. P values less than 0.05 were considered statistically significant.

## Results

### Mesenchymal Stem Cell Culture and Identification

**Figure 1:**
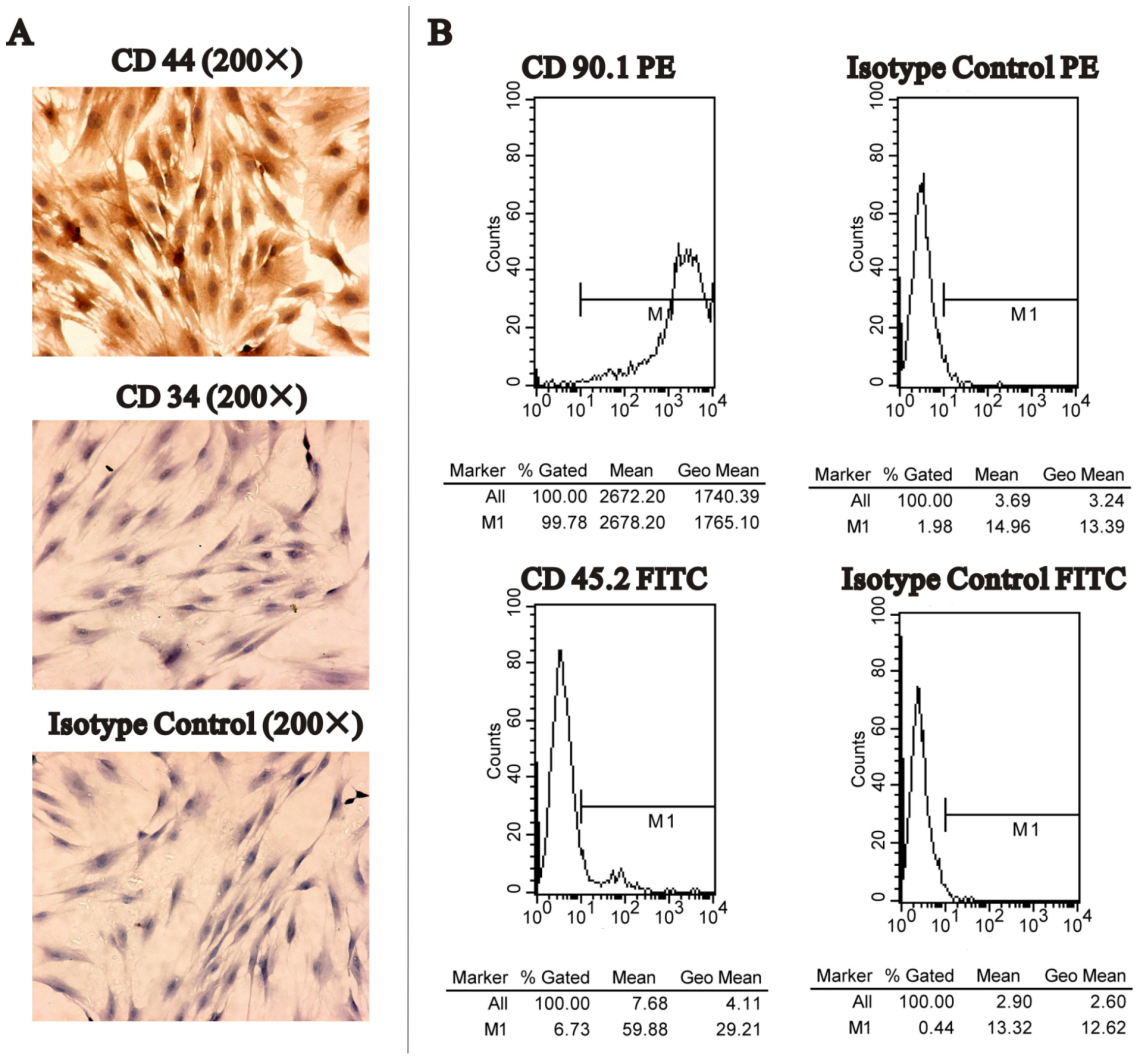
Detection of surface markers of MSCs. A. Immunocytochemistry results showed that MSCs were positive for CD44 (upper), and negative for CD34 (middle) and isotype control (lower). B. Flow cytometry analysis of MSCs. Almost the entire tested MSCs showed positive for CD90 (left upper), and negative for CD45 (left lower), isotype control Ig G2 PE (right upper) and isotype control Ig G1 FITC (right lower).

Most of the cells collected from bone marrow rinses by centrifuge were mononuclear MSCs. The primary cells were circular and began to stick after 24 h, and the morphology of cells changed to spindle or short rod-like in 3–4 days. About 1 week later, the cells formed colonies and were arranged like radial or concentric circles. They grew to 80%–90% confluence on days 7–10 after initial plating and had a definition of passage 0. Cells between passages three and ten were maintained in a spindle-shaped and fibroblast-like form and were used for experiments. Immuno-cytochemistry and flow cytometry showed most of the cells were positive for CD44 and CD90 but negative for CD34 and CD45 (Fig. 1). The osteogenic differentiation and adipogenic differentiation capacity of MSCs have been confirmed by our group previously [33]. These MSCs immunophenotype profiles agreed well with previously reported expression patterns of surface antigens using rMSCs [28].

### Strong Correlations between Reporter and Therapeutic Genes Indicated by qRT-PCR and Immunofluorescence

**Figure 2:**
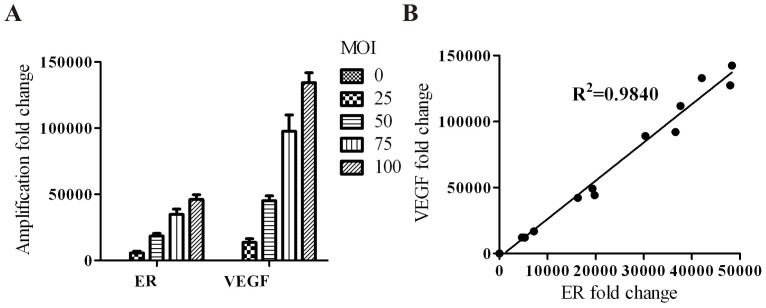
hERL and VEGF165 expression in MSCs detected by qRT-PCR. A revealed the relative expression of hERL and VEGF165 increased with adenovirus titer. Very low expression of both genes was seen in the control nontransfected group (MOI = 0), and VEGF165 gene expression showed stronger than that of the hERL gene. B presented the high correlation of the expression of hERL and VEGF165 (R^2^ = 0.9840).

**Figure 3:**
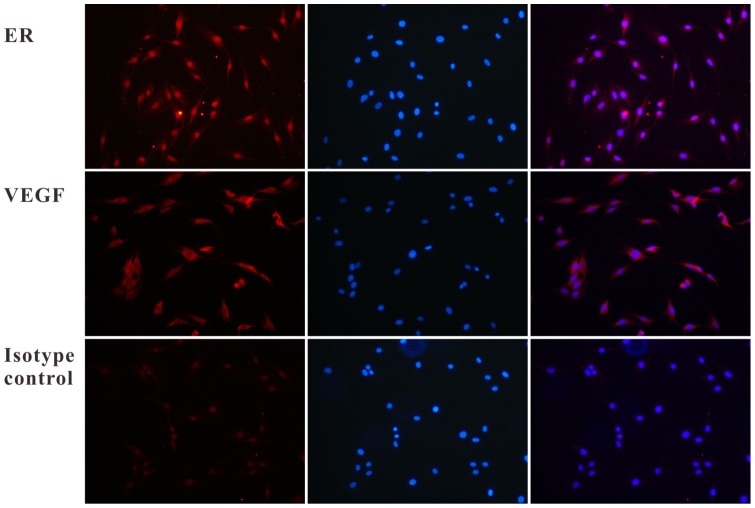
Immunofluorescence results for hERL and VEGF165 expression in Ad-EIV-MSCs (200×). The upper row showed an intense/moderate immunoreactive signal in the peri-nuclear/nuclear regions, which suggested the expression of the estrogen receptor-α subtype. The image on the left was the positive expression of ER, and the middle one was the nucleus staining under the same field of vision. The image on the right was the overlay image, which showed the sub-cellular localization of ER clearly. The middle row showed an intense immune-reactive signal of VEGF165, which was detected in the cytoplasm. The order of the image was same as the upper row. The third row was the isotype control result.

The results of qRT-PCR showed an obvious mRNA expression of hERL and VEGF165 in Ad-EIV-MSCs, while the control nontransfected MSCs revealed low hERL and VEGF165 mRNA expression (Fig. 2 A). Over the range of viral titers tested, a high correlation (R^2^ = 0.9840, P<0.05) existed between the expressions of the two linked genes (Fig. 2 B). Figure 3 shows an immunofluorescence image that exhibited protein expression of hERL and VEGF165. From the PCR and immunofluorescence results, the mRNA and protein expression of VEGF165 was stronger than that of hERL. These results demonstrated successful transduction of the reporter and therapeutic gene at the same time, and the reporter gene could reflect the therapeutic gene indirectly.

### Cellular Uptake of ^18^F-FES

**Figure 4:**
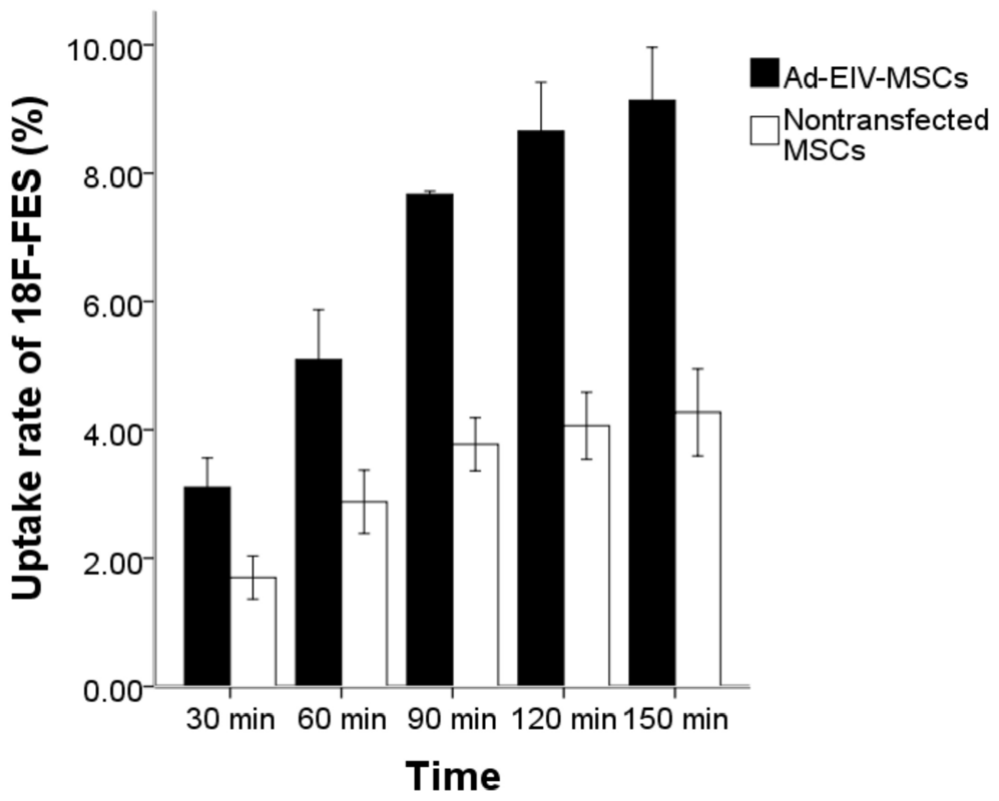
Time-dependent cellular uptake experiment. Data are means and standard deviations from three independent experiments.

Figure 4 demonstrated a time-dependent increase in the accumulation of ^18^F-FES in both the Ad-EIV-MSCs (MOI = 100) and control nontransfected MSCs, and the highest uptake rate occurred at 150 min, with the peak values of 9.13%±0.33% (n = 3) and 4.27%±0.27% (n = 3), respectively. The results obtained from the control group showed a remarkable difference compared with Ad-EIV-MSCs at each time point (t = 10.574–40.260, P<0.01), which supported the specificity of ^18^F-FES for hERL.

### *In Vivo* Micro-PET/CT Imaging

**Figure 5:**
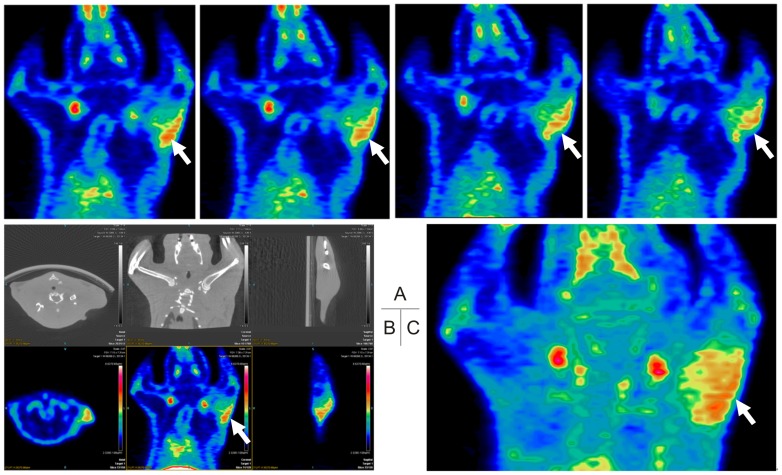
Micro-PET/CT images of a representative animal. ^18^F-FES PET/CT scan was performed to detect hERL expression in the left forearm of rat. Intense ^18^F-FES uptake (arrows) was observed at the left forearm, while no uptake of ^18^F-FES at the contralateral forearm. (A. Coronal slices of an animal's PET imaging; B. The transverse, coronal and sagittal images of CT (upper) and PET (lower) images; C. Image of maximum intensity projection.).

Much higher radioactivity could be seen in the left foreleg where Ad-EIV-MSCs were injected than that in the opposite foreleg, which served as self-control (Fig. 5). Identical ROIs were drawn on the left and right forelegs, and the uptake values were 0.46±0.11% ID/g and 0.11±0.03% ID/g (n = 4), respectively (P<0.05).

## Discussion

In order to evaluate the success of stem cell transplantation and to monitor the expression of therapeutic genes in living individuals, development of noninvasive imaging modalities capable of identifying the location, magnitude and duration of cellular survival and fate is required [27]. One of best techniques available is radionuclide reporter gene imaging, which enables accurate non-invasive monitoring of therapeutic genes and cells, quantitatively and repeatedly.

Recently, investigators have developed a reporter gene PET tracer system, hERL/^18^F-FES, for monitoring gene therapy noninvasively [2], [10], [34]. Plasmid or adenovirus was used as the carrier of the reporter gene; human thymidine phosphorylase was used as a therapeutic gene and uptake of [^3^H] estradiol demonstrated specific uptake in infected cells. In the present study, MSCs were used as the platform for gene therapy and recombinant adenovirus Ad5-hERL-IRES-VEGF was used as the vector. The aim was to evaluate the feasibility of a new reporter gene/probe system hERL/^18^F-FES for monitoring gene and cell therapy; the ultimate goal is to monitor MSCs and VEGF165 gene therapy in vivo. This is the first report on the novel reporter gene system hERL/^18^F-FES being used for monitoring cell/gene therapy in vivo. We demonstrated its feasibility from our primary in vitro and in vivo results, which may provide a good foundation for further study.

In order to achieve gene therapy, a carrier or ‘vector’ is required to deliver the therapeutic genetic material into the special target cells. Different gene delivery systems with various favorable characteristics have been developed. Adenovirus is still the preferred gene delivery vector owing to its high efficiency [2], [35], [36]. This is especially true in humans where high levels of short-term gene expression, such as for therapeutic angiogenesis, are required. Compared with other cell types considered for cardiomyopathy, MSCs appear to possess unique properties that may allow for convenient and highly effective cell therapy. MSCs can be used allogenetically and delivered systemically, and can differentiate into many cell types in the proper microenvironments. Furthermore, MSCs can be readily transduced by a variety of vectors and maintain transgene expression after in vivo differentiation [27].

In the initial in vitro studies, our data showed that the reporter gene hERL and the therapeutic gene VEGF165 were successfully transfected into MSCs through the recombinant adenoviruses vector Ad5-hERL-IRES-VEGF. These two genes co-expressed and correlated well with each other. These findings indicated that the adenovirus was a good vector with high transfect efficiency and provide a theoretical basis for animal research in vivo. In the cellular uptake experiment, uptake rates of ^18^F-FES in Ad-VIE-MSCs at all time points were much higher than those of nontransfected MSCs. However, a small but detectable rise in uptake was also seen in the nontransfected MSCs. It may be related to the non-specific uptake or conglutination of ^18^F-FES, and this non-specific uptake increased over time. Moreover, nontransfected MSCs perhaps have a very low expression of ER.

In the primary in vivo study, we injected virus infected MSCs into rat foreleg muscle and used micro-PET/CT imaging for evaluation. After intravenous injection, ^18^F-FES has been shown to distribute rapidly in the whole body, including the brain. It is also washed out rapidly, with the exception of ER-expressing tissue and tissues such as the liver, kidney, bladder and intestines, for these organs are the primary organs involved in ^18^F-FES metabolism and excretion [16], [37]. Positive accumulation of ^18^F-FES localized in the left foreleg was observed. The region of high ^18^F-FES uptake indicated hERL expression. Some bone uptakes were observed, which indicates either defluorination of ^18^F-FES in vivo or ^18^F- impurity presenting in the probe. The biodistribution of ^18^F-FES observed using micro-PET imaging in the present study was similar to that reported in previous studies [38], [39].

The advantage of our study was that the PET probe ^18^F-FES is a radiopharmaceutical already being used for human studies, which can access a wide range of tissues, including the brain. In addition, because the reporter hERL gene lacks a DNA binding domain it can no longer work as a transcription factor, and has no physiological function. Thus, it's possible to obtain images of the therapeutic gene and stem cells through the reporter gene indirectly. All of these features could facilitate easier translation of our system from the bench to bedside.

Our study had some limitations that should be acknowledged. First, our recombinant adenovirus did not contain the fluorescence (GFP or RFP) gene, so we could not observe transfection efficiency using the fluorescence microscopy which is very convenient. Second, our current study lacked a radio-ligand receptor binding assay. Unlabeled estradiol should have been used to block the estrogen receptor and test non-specific binding. A competitive binding assay is also needed.

Although further studies, such as those involving a receptor binding assay and in vivo animal models are still needed, the positive in vitro results and proof-of-concept in vivo images obtained in our study using a rat muscle model indicated that our new reporter gene/reporter probe system is potentially applicable for monitoring gene/cell therapy.

## Conclusion

In summary, our study demonstrated that Ad5-hERL-IRES-VEGF was a good vector for the reporter gene hERL and the therapy gene VEGF165. Cultured MSCs infected by Ad-EIV expressed therapeutic and reporter genes simultaneously, and accumulated the radioligand ^18^F-FES specifically. Successful micro-PET/CT imaging of the rat left foreleg injected with Ad-EIV-MSCs demonstrated the efficacy of utilizing hERL as reporter gene, and ^18^F-FES as PET probe for monitoring gene and cell therapy in vivo. These findings demonstrated that hERL/^18^F-FES is feasible for monitoring gene/cell therapy and provided sufficient evidence to warrant further studies.
